# Pitfalls in the detection of cholesterol in Huntington’s disease models

**DOI:** 10.1371/505886e9a1968

**Published:** 2012-10-11

**Authors:** Manuela Marullo, Marta Valenza, Valerio Leoni, Claudio Caccia, Chiara Scarlatti, Agnese De Mario, Chiara Zuccato, Stefano Di Donato, Ernesto Carafoli, Elena Cattaneo

**Affiliations:** Centre for Stem Cell Research, Università degli Studi di Milano, 20133 Milan, Italy; Centre for Stem Cell ResearchUniversità degli Studi di Milano; Foundation IRCCS Insitute of Neurology Carlo Besta; IRCCS-Istituto Nazionale Neurologico Carlo Besta; Venetian Institute for Molecular Medicine; Venetian Institute for Molecular Medicine; University of Milan; IRCCS Istituto Neurologico Carlo Besta; Venetian Institute for Molecular Medicine; Department of Pharmacological Sciences and Centre of Stem Cell Research, University of Milan

## Abstract

Background Abnormalities in brain cholesterol homeostasis have been reported in Huntington’s disease (HD), an adult-onset neurodegenerative disorder caused by an expansion in the number of CAG repeats in the huntingtin (HTT) gene. However, the results have been contradictory with respect to whether cholesterol levels increase or decrease in HD models. Biochemical and mass spectrometry methods show reduced levels of cholesterol precursors and cholesterol in HD cells and in the brains of several HD animal models. Abnormal brain cholesterol homeostasis was also inferred from studies in HD patients. In contrast, colorimetric and enzymatic methods indicate cholesterol accumulation in HD cells and tissues. Here we used several methods to investigate cholesterol levels in cultured cells in the presence or absence of mutant HTT protein. Results Colorimetric and enzymatic methods with low sensitivity gave variable results, whereas results from a sensitive analytical method, gas chromatography-mass spectrometry, were more reliable. Sample preparation, high cell density and cell clonality also influenced the detection of intracellular cholesterol. Conclusions Detection of cholesterol in HD samples by colorimetric and enzymatic assays should be supplemented by detection using more sensitive analytical methods. Care must be taken to prepare the sample appropriately. By evaluating lathosterol levels using isotopic dilution mass spectrometry, we confirmed reduced cholesterol biosynthesis in knock-in cells expressing the polyQ mutation in a constitutive or inducible manner. *Correspondence should be addressed to Elena Cattaneo: elena.cattaneo@unimi.it

## Introduction

Huntington’s disease (HD) is an adult-onset neurodegenerative disorder caused by an expansion in the CAG repeat in the 5’ terminus of the HD gene 1. In the Huntingtin protein (HTT), which is a ubiquitously expressed protein with beneficial roles in brain neurons, the CAG repeat is translated into a polyglutamine (polyQ) tract 2. HD pathology involves multi-faceted mechanisms that affect nearly all aspects of cellular physiology 2. Among these mechanisms, abnormalities in cholesterol metabolism have been reported in HD cellular and animal models and in tissues from HD patients 3, 4. In particular, mRNA levels of key genes involved in cholesterol biosynthesis are reported to be reduced in clonal striatal-derived cells that overexpress mutant HTT (muHTT) 5 as well as in brain samples from HD mice and in post-mortem HD cerebral specimens 6. Biochemical and mass spectrometry analyses confirmed reduced levels of cholesterol precursors early in the disease process in brains from R6/2, YAC46, YAC72 and YAC128 knock-in mice and transgenic rats. At later time points, the same animals showed decreased levels of sterols/cholesterol 6, 7, 8. Brain cholesterol homeostasis is also affected in humans in the early stages of the disease 9, 10. One possible mechanism that could explain this is a reduction in the nuclear translocation of SREBP, a master transcriptional activator of several cholesterogenic genes 6. A recent report confirmed a reduced cholesterol level in neural stem (NS) cell lines developed from embryonic brains of wild-type and knock-in HD mice expressing full-length endogenous normal HTT (Hdh^7/7Q^) or muHTT (Hdh^140/140Q^) but not in NS cell lines derived from embryonic stem cells of heterozygous knock-in HD mice (Hdh^140/7Q^) 11. In contrast, other groups reported cholesterol accumulation, not cholesterol reduction, in mouse and human HD tissues and cell cultures 12, 13, 14. In particular, it was reported that muHTT could interact with caveolin-1 and cause an accumulation of intracellular cholesterol in primary neurons and in brains from the YAC72 transgenic mouse model of HD 12. Further, primary rat neurons that overexpress muHTT fragments showed sterol accumulation, and SIRT2 inhibition reduced sterol levels via decreased nuclear trafficking of SREBP2 13. Lastly, muHTT expression resulted in the accumulation of cholesterol in HD cellular and murine models and in HD-affected human brains, and its altered cellular distribution contributed to NMDA-mediated excitotoxicity 14.

These conflicting data, which might be the result of the use of different techniques and experimental materials in the different studies, raise the questions of whether cholesterol levels increase or decrease in HD and how this affects cellular and brain dysfunction in the disease. A tentative unifying hypothesis suggests that decreased cholesterol biosynthesis in HD may be the consequence of sterol accumulation 4, but further clarification regarding cholesterol level changes in HD is needed.

In this study, we used several methods to measure sterols and cholesterol levels in immortalized knock-in cells derived from the embryonic striatum of mice carrying the endogenous HTT gene with 7Q (ST Kin^7/7Q^) or 109Q inserted into the mouse locus (ST Kin^109/109Q^). We found different results depending on the technique adopted and that definitive methodology, such as gas chromatography-mass spectrometry (GC-MS), should be favoured. Changes in cholesterol levels have been linked to NMDA receptor activity 14, but we could not detect this receptor when analysing the same cells used in the earlier study. Our data also reveal that sample preparation, degree of confluence and clonal properties strongly influence the results and, in particular, the level of intracellular cholesterol that is detected. A further confounding factor may stem from changes in the properties or technical issues related to the distribution and/or culture of target cell lines carrying the HD gene.

## Results and discussion

****Comparison of different methods that measure sterol content****

A number of methods are available for measuring lipid and cholesterol content in cells. Among the commercially available kits, the enzymatic method, which is based on cholesterol esterase and oxidase, has been used to measure cholesterol in HD samples 6, 13, 14. This method is not specific for cholesterol but all sterols can be measured since the β-OH group involved in the enzymatic reaction mediated by cholesterol oxidase is common to many sterols. To quantify the extent of this possible confounding effect, we loaded ST Kin^7/7Q^ cells with increasing concentrations of exogenous cholesterol and then subjected the cellular preparations to lipid detection by the enzymatic method.

Specifically, ST Kin^7/7Q^ cells were cultured in normal growth medium and incubated with increasing concentrations of cholesterol (0, 5, 15, 30, 50 and 80 µg/ml) for 16 hours (Fig. 1). Lipids were then purified by solvent-based extraction and measured with the Amplex® Red Enzyme Assay (Invitrogen). Figure 1b shows that the enzymatic method detected the accumulation of exogenous cholesterol in ST Kin^7/7Q^ cells loaded with 5 µg/ml and 15 µg/ml cholesterol, while linearity was lost at 30 µg/ml. Higher doses of cholesterol were toxic, as evident in the phase contrast pictures of cultured cells treated with 50 µg/ml and 80 µg/ml cholesterol (Fig. 1a).

These findings confirmed that the enzymatic method could reliably detect accumulation of exogenously supplied cholesterol in cultured cells (up to 15 µg/ml). In addition, the enzymatic detection may give misleading results when measuring subtle changes in endogenous cholesterol levels as this assay reflects the total sterol rather than the total cholesterol content, as noted previously 15.

Colorimetric methods (filipin staining and Nile Red staining) and gas chromatography-mass spectrometry (GC-MS) have also been used to detect cholesterol levels. The colorimetric methods are based on fluorescent molecules that bind to lipids and sterols with high affinity, while GC-MS is an analytical method that allows detection of cholesterol after its chromatographic separation from other sterols and by isotope dilution with deuterium labelled internal standard. To compare the analytical sensibility of these methods, we performed cholesterol loading or depletion of ST Kin^7/7Q ^cells and then measured the cholesterol content using both methods.

In the first set of experiments, ST Kin^7/7Q ^cells were incubated with 0 (vehicle), 3, 5, 7 and 10 µg/ml of cholesterol for 16 hours and then analysed by filipin staining or GC-MS. As shown in Figure 1c, visual inspection of the stained cultures revealed a difference in the filipin staining of cells cultured with 10 µg/ml of cholesterol compared to control, but we could not detect any differences in cultures treated with <7 µg/ml cholesterol (Fig. 1c; Suppl. Fig. 1a). Notably, the fluorescence intensity varied from field to field for the 10 µg/ml culture, leading to a large SD (Suppl. Fig. 1a). GC-MS analysis showed a gradual dose-dependent increase of cholesterol content in ST Kin^7/7Q ^cells (Fig. 1d).

Next we exposed cultures of ST Kin^7/7Q ^cells to increasing concentrations of methyl-beta-cyclodextrin (MβCD; 0, 1, 5, 10 and 25 mM) for 1 hour in order to deplete cells of cholesterol. The cholesterol content was then measured by filipin staining or by GC-MS. Figure 1e and 1f show that the cholesterol depletion was detectable by both methods. However, GC-MS was more sensible compared to filipin staining. Specifically, when cells were treated with 1 mM MβCD, the decrease in cholesterol content was detectable by GC-MS but not by filipin staining. We concluded that GC-MS, but not filipin staining, could detect increased cholesterol levels in vitro on the order of a few µg/ml. In addition, filipin staining resulted in variable fluorescence within the same sample. GC-MS was also more sensible for detecting decreased cholesterol levels in vitro, because GC-MS, but not filipin staining, detected a decrease in cholesterol after acute treatment with >1 mM MβCD. The higher MβCD doses decreased cell survival. We concluded that although filipin can detect free cholesterol, it is not quantitative and may be more suitable for detection of cholesterol *in situ*. In contrast, GC-MS is reliable when appropriate standards are used.

**Cholesterol levels in ST Kin7/7Q cells loaded with or depleted of cholesterol.**
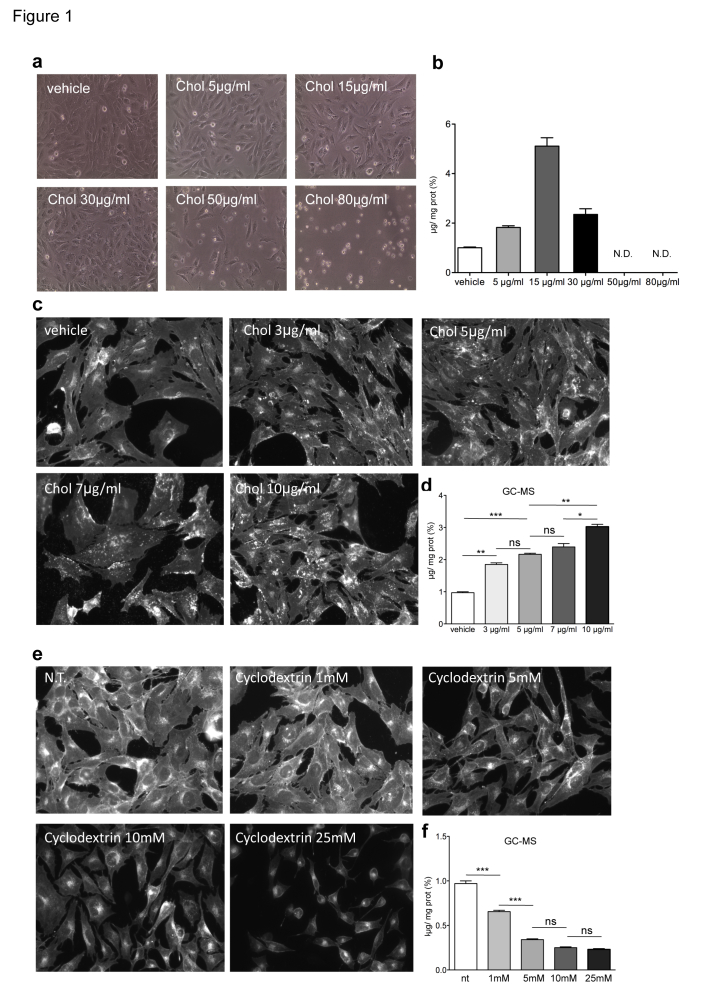
**a)** Phase contrast images of ST Kin^7/7Q^ cells loaded with increasing concentrations of cholesterol (5, 15, 30, 50 and 80 µg/ml) show that high concentrations of cholesterol (50–80 µg/ml) are toxic to the cells and lead to dramatic morphological changes. 10X magnification. **b)** Cholesterol levels, as measured by enzymatic method a, in the same ST Kin^7/7Q^ cells shown in (a), show that cholesterol accumulation is detected in ST-Kin7/7Q cells loaded with 5 and 15 µg/ml cholesterol, while linearity is lost at 30 µg/ml. In the cells loaded with the highest amounts of cholesterol, i.e. 50 and 80 µg/ml, we could not measure cholesterol accumulation because these doses led to cell death, as shown in the phase contrast images. **c-d)** Comparison of cholesterol quantification using filipin staining (c) and GC-MS (d) in ST Kin^7/7Q^ cells loaded with increasing concentrations of cholesterol (3, 5, 7 and 10 µg/ml) suggests that filipin staining is less sensitive than GC-MS. The images shown were cropped from 20X magnification images. **e-f)** Comparison of filipin staining (e) and GC-MS (f) quantification of cholesterol in ST Kin^7/7Q^ cells depleted of cholesterol by exposing them to increasing concentrations of MβCD (1, 5, 10 and 25 mM). The images shown were cropped from 20X magnification images. Cells were plated at the same densities in 6-well plates for cholesterol detection by the enzymatic method and by GC-MS and in 12-well plates for filipin staining. Two independent experiments were performed in which the cells were processed in parallel for filipin staining and for cholesterol quantification by GC-MS. The graphs (d, f) represent the mean ± SEM as determined from the analysis of two independent experiments in which each sample was processed in duplicate. Statistics: One-way ANOVA and the Newman-Keuls Multiple Comparison Test were performed. Significant differences are indicated by asterisks: *p < 0.05; **p < 0.01; ***p < 0.001).

**Sterol content in striatal immortalized knock-in cells expressing muHTT**

To investigate the consistency of colorimetric staining, enzymatic methods and GC-MS for detecting sterol changes in HD cell models, we evaluated intracellular lipid and cholesterol levels in ST Kin^7/7Q ^cells and in ST Kin cells expressing muHTT with 109Q (ST Kin^109/109Q^) 16. We first used Nile Red, a vital lipophilic dye that is used to label neutral lipids (i.e. triacylglycerols or cholesteryl esters) 17, to detect the accumulation of intracellular lipid droplets in ST Kin^7/7Q^ cells and in two ST Kin^109/109Q^ cell clones (clone #2 and clone #6). As shown in Figure 2a, low-magnification fluorescent images did not show differences in lipid accumulation between wild-type and mutant cells. Notably, the staining was heterogeneous within each cell line; thus, high-magnification fluorescent analysis of a few cells or single cells was avoided, as this would lead to misleading results.

In addition to Nile Red, we also tried filipin staining. We did not find any significant differences between the staining of ST Kin^7/7Q^ cells and the staining of ST Kin^109/109Q^ clones #2 or #6 (Fig. 2a). Filipin staining was heterogeneous in that some cells showed a punctuate signal and some cells showed more diffuse staining. In addition, there was high variability in the fluorescent intensity in different fields of the same well (Suppl. Fig. 1b). This variability was more evident within the different clones compared to in experiments in which cholesterol was added or removed (Fig. 1c, e). Of note, we found that filipin exhibited rapid photo-bleaching and only modest natural fluorescence under UV excitation, which introduces another confounding factor in the detection of cholesterol using this method.

These results using two qualitative colorimetric methods, i.e. Nile Red and filipin staining, indicated that the levels of neutral lipids and free cholesterol were similar in wild-type and muHTT cell lines, conflicting with the results reported by other researchers that have used the same cell lines 14. Indeed, in the study by Del Toro et al. (2010), the authors detected increased Nile Red and filipin staining, as well as cholesterol accumulation as detected by the enzymatic method, in a clone of ST Kin^109/109Q^ cells compared to ST Kin^7/7Q^ cells 14.

We are aware that filipin staining cannot be considered a quantitative method for measuring sterol/cholesterol content in cells or tissues, so we rechecked the levels of intracellular sterols in ST Kin^7/7Q^ cells and ST Kin^109/109Q^ cells by the enzymatic method used by Del Toro et al. (2010) 14 and in our previous study 6. Notably, different protocols for the preparation of the samples were used in the two studies. The protocol used by Del Toro et al. (2010) 14 was based on a protein lysate preparation followed by cholesterol esterase and oxidase incubation (method a). In Valenza et al. (2005) 6, a solvent-based extraction was used to obtain a lipid fraction from the cells (method b).

In agreement with the filipin staining results shown in Figure 2a, the sterol content as measured by method a was the same in ST Kin^7/7Q^ and in one clone of ST Kin^109/109Q^ cells (clone #2, Fig. 2B) but was increased in the second ST Kin^109/109Q^ cell clone (clone #6). Because sterols are lipids, it is better to measure sterols in a cellular lipid extract rather than in a cellular protein extract. To evaluate whether the preparation of the sample influenced cholesterol/sterol detection, we also measured sterol levels after lipid extraction from the same samples (method b; Fig. 2). As shown in Figure 2c, method b, but not method a, detected similar sterol levels in ST Kin^7/7Q^ cells and in the two ST Kin^109/109Q ^cell clones (Fig. 2B). In Del Toro et al. (2010), the finding of an increased level of cholesterol at the plasma membrane was linked to increased sensitivity of the muHTT cells to NMDA-mediated excitotoxicity 14. However, we failed to detect the activity of NMDA or AMPA receptors, as judged by Ca2+ efflux (Suppl. Fig. 2a, b) and by a lack of change in the expression of the NMDA 2B, GLUR1 and GLUR5 genes by qualitative PCR (data not shown) in ST Kin^7/7Q^ cells. Moreover, we also failed to detect the expression of the NMDA subunit NR1, as judged by real time qPCR, in ST Kin^7/7Q^ and ST Kin^109/109Q^ cells (Fig. 2S c).

To further verify the level of cholesterol in ST Kin^7/7Q^ and ST Kin^109/109Q^ cells, we analysed our cellular preparations by isotope dilution GC-MS. The data in Figure 2d confirmed the results obtained using the enzymatic method on lipid extracts (method b) and previous data from an immortalized striatal cell line with inducible expression of the N-terminal fragment of muHTT 6. Specifically, we detected no changes in cholesterol levels in this cell system growing in serum-containing medium.

Taken together, these findings suggest that differences in sample preparation methods, such as the use of lipid vs. protein extracts, can lead to differences in the detection of changes in cholesterol levels. This may account for the inconsistent results from different laboratories when different methods are used. Our results also highlighted the importance of measuring cholesterol in cells and tissues with the most appropriate method; specifically, GC-MS appears to be the most sensitive and reliable method. In agreement with this finding, all results obtained by GC-MS in different studies show decreases in cholesterol levels in HD cells and tissues 7, 8, 11. Finally, although outside the scope of the present study, our inability to detect the NMDA-AMPA receptor in ST Kin cell lines made it almost impossible to study the possible link between cholesterol changes and increased sensitivity to NMDA-mediated excitotoxicity of these muHTT cells, as proposed previously 14.

**Neutral lipids and sterols in ST Kin 7/7Q and in ST Kin109/109Q cells.**
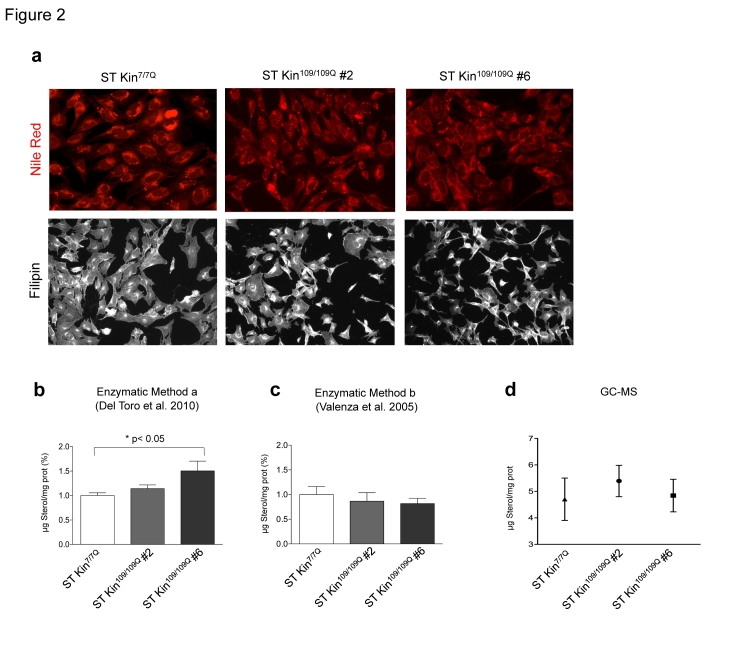
a) Staining with Nile Red, a marker of neutral lipids, does not show any differences between wild-type cells (ST Kin^7/7Q^) and HD cells (ST Kin^109/109Q^, clone #2 and clone #6). Similarly, filipin staining does not show any differences between the cell lines. 20X magnification. b-d) Comparison of two enzymatic methods and GC-MS for measuring cholesterol levels in ST Kin^7/7Q^ and ST K-in^109/109Q^ cell lines. The two methods differ in the sample preparation method that is used prior to cholesterol detection. The cells were processed to obtain either a protein lysate, as described in Del Toro 2010 (method a), or a lipid extract, as described in Valenza et al. 2005 (method b), and both preparations were subjected to cholesterol esterase and oxidase assay. Using enzymatic method a, an increase in sterol levels was detected in ST Kin^109/109Q^ clone #6 compared to wild-type ST Kin^7/7Q^ cells, while similar levels of sterols were found in ST Kin^109/109Q^ clone #2 and ST Kin^7/7Q^ cells (b). Sterol quantification by method b does not reveal any differences in sterol content in ST Kin^7/7Q^ versus ST Kin^109/109Q^ clonal cells (c). Similarly, GC-MS showed no differences between the wild-type and HD cell lines (d). Each sample was analysed in duplicate, and the graphs show the mean ± SEM of three independent experiments. Statistics: One-way ANOVA and the Newman-Keuls Multiple Comparison Test were performed. Significant differences are represented by asterisks: *p < 0.05.

**Levels of cholesterol precursors in ST Kin cells that express muHTT**

To verify the decrease of the cholesterol levels in the HD cells used in this study, we used isotope dilution GC-MS to measure cholesterol precursors, including lanosterol, lathosterol, desmosterol and 7-dehydroxycholesterol (7DHC). Cells were collected when they reached 80–90% confluence. Figure 3 shows that the levels of lanosterol and lathosterol in both ST kin^109/109Q^ clones were unchanged compared to control cells, while the levels of desmosterol and 7DHC were significantly decreased in both ST Kin^109/109Q^ clones compared to control cells (Fig. 3 c-d). These results indicate a reduction in the availability of some, but not all, cholesterol precursors in these HD cells.

Cholesterol homeostasis *in vitro* might be influenced by the culture conditions, including cell over-confluence 18. To evaluate whether cell density affected cholesterol detection, we measured the levels of cholesterol and cholesterol precursors in ST Kin^7/7Q^ cells and in both clones of ST Kin^109/109Q^ cells that were grown to over-confluence. For these experiments, rather than splitting the cells after they were confluent, they were left for another 2 days in culture, leading to ~40–50% over-confluence. In contrast to findings obtained in sub-confluent growth conditions (Fig. 2d), cholesterol was found to be increased in ST Kin^109/109Q^ clone #6 cells compared to ST Kin^7/7Q^ cells (Fig. 4a). Conversely, the cholesterol content was similar in ST Kin^7/7Q ^cells and ST Kin^109/109Q^ clone #2 cells (Fig. 4a). The lanosterol level was unchanged in both clones (Fig. 4b), while lathosterol was significantly increased only in ST Kin^109/109Q^ clone #6 cells compared to ST Kin^7/7Q ^cells (p<0.01) (Fig. 4c). Moreover, the levels of desmosterol and 7DHC were significantly increased in ST Kin^109/109Q^ #6 compared to ST Kin^109/109Q^ clone #2 cells (p<0.01) and compared to ST Kin^7/7Q ^cells (Fig. 4d-e). These results suggest that growth conditions may affect the levels of different sterols in HD clones in different ways and that these effects are unlikely to be linked to the presence of muHTT.

**Levels of cholesterol precursors in ST Kin7/7Q and ST Kin109/109Q cells.**
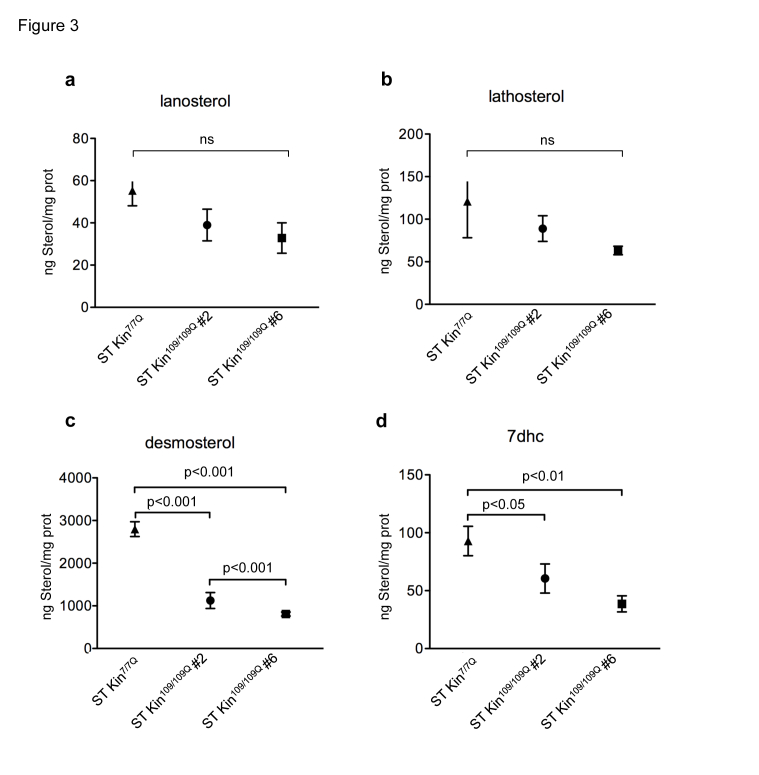
Isotopic dilution GC-MS performed on ST Kin^7/7Q^ and ST Kin^109/109Q^ cells grown in standard fetal bovine serum-containing medium shows that a-b) lanosterol and lathosterol are moderately reduced in clone # 2 and #6 compared to wild-type cells, c-d) while downstream cholesterol precursors (desmosterol and 7dhc) are significantly reduced in the muHTT clones compared to wtHTT cells. The results are shown as the mean ± SEM of three independent experiments. Statistics: One-way ANOVA and the Newman-Keuls Multiple Comparison Test were performed.

Because we obtained different results in ST Kin^109/109Q^ clone #2 and clone #6 cells, we next used mass spectrometry to measure the levels of cholesterol and cholesterol precursors in immortalized striatal cells that express the N-terminal fragment of muHTT upon doxycycline administration 5. This model allows us to measure the levels in the same cellular background with or without the expression of muHTT and to investigate differences that are linked to muHTT expression. Importantly, this circumvents clone-dependent effects. In agreement with results obtained previously using the enzymatic method 6, we found that the cholesterol level did not vary significantly in the uninduced cells compared to cells that were induced to express muHTT (33.60±2.14 μg chol/mg prot vs. 33.86±1.76 μg chol/mg prot, respectively). These cells were grown in serum-containing medium, and the exogenous cholesterol sources present in the medium may have helped the cells maintain a constant level of cellular cholesterol. On the other hand, lathosterol, which is an indicator of cholesterol biosynthesis, was reduced in cells expressing muHTT compared to cells cultured in uninduced conditions (1.647±0.1048 ng latho/mg prot vs. 2.841±0.2988 ng latho/mg prot, respectively; p<0.01).

**Levels of cholesterol precursors in over-confluent ST Kin7/7Q and ST Kin109/109Q cells.**
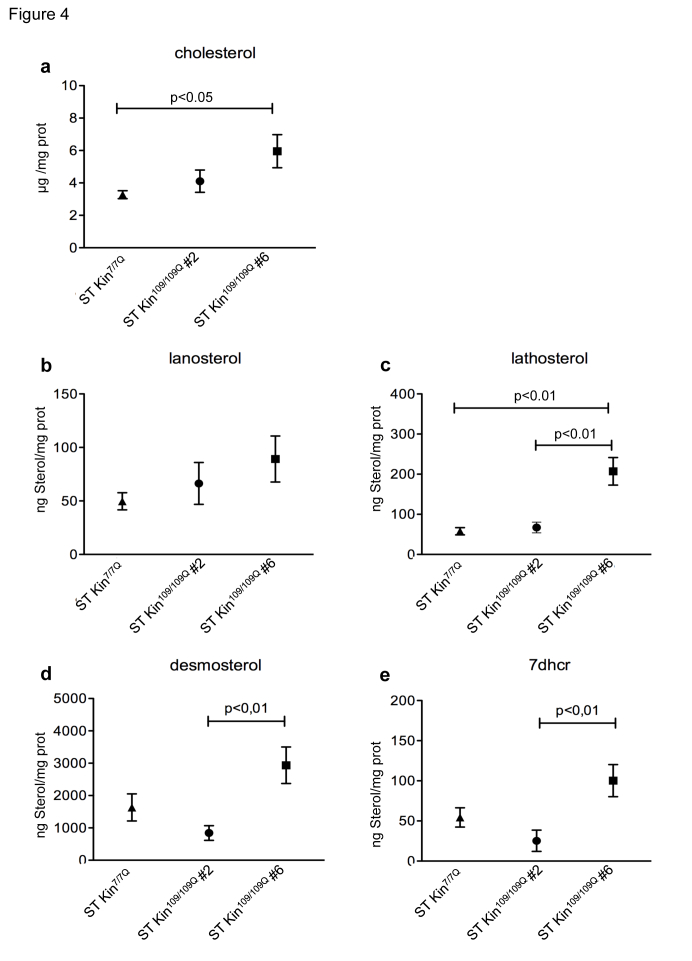
The same mass spectrometry analysis of cholesterol and cholesterol precursors reported in Figure 2 was also performed in cells harvested at over-confluence. a) Cholesterol levels are similar in ST Kin^109/109Q^ clone #2 and ST Kin^7/7Q^ but are increased in ST Kin^109/109Q^ clone #6 compared to ST Kin^7/7Q^. b) Lanosterol was not significantly different in the mHTT clones and wild-type cells, while lathosterol (c) was higher in ST Kin^109/109Q^ clone #6 compared to wild-type and ST Kin^109/109Q^ clone #2. d-e) Similar changes are observed in the downstream cholesterol precursors desmosterol and 7dhc. Although it does not reach the significance, desmosterol and 7dhc are increased in ST Kin^109/109Q^ clone #6 compared to ST Kin^7/7Q^. There are no significant differences between ST Kin^109/109Q^ clone #6 and ST Kin^7/7Q^ cells. The results are shown as the mean ± SEM from analyses in two independent experiments. Statistics: One-way ANOVA and the Newman-Keuls Multiple Comparison Test were performed.

Taken together, these results illustrate the complexity of detecting cholesterol and its precursors *in vitro* and show that clonal variability and differences in cell density can lead to considerably different results.

## Conclusions

Cholesterol is a structural component of biological membranes and is the precursor of numerous signalling molecules. In the brain, cholesterol is involved in neurite outgrowth, myelination and synaptic activity, and its importance in brain physiology and pathology is clear from neurological diseases in which there are changes in its level or distribution 3, 4. Changes in sterol levels in cellular and animal models of HD have been detected in previous studies, but the direction of these changes remains a matter of debate. Specifically, while some studies report a decrease in cholesterol levels in HD models 6, 7, 8, 11, 15, others report the opposite 12, 13, 14.

In this study, we investigated factors that could underlie these conflicting findings.

As we expected, the methods used to detect cholesterol, sample preparation methods, cell line clonal properties and cell growth conditions all influenced the levels of cholesterol that were detected. It is critical to be aware of the limitations and artefacts that arise from using less sensitive methods or from inappropriate sample preparation. Colorimetric methods such as filipin staining should be avoided when the aim is quantitative detection of cholesterol levels in cells or tissue samples. Cholesterol levels may be different due to the existence of variants of what is believed to be the same cell line. In this study, we found that two clones of ST Kin cells, which were derived years ago in our laboratory at the University of Milan 16, were not, in fact, identical. The clones have been distributed by us and by collaborating laboratories to many other laboratories, as well as by direct transfer from a recipient lab to yet another lab. Different laboratory practices and mislabelling may have resulted in the propagation of cells with properties that are different from expected or that differ from lab to lab. Contrary to reports from other groups 14, 19, we found no NMDAr or AMPAr activity and no expression of the NMDA subunit NR1 mRNA in ST-Kin cells. Genetic-based assays for the identification of cell lines 20 and the presence of a dedicated biorepository that controls the quality and distribution 21 of cell lines would reduce the risk of laboratory errors; such issues have already caused major damage in other fields 22 23.

When a novel disease target emerges, prior to embarking in drug development initiatives, it is essential to validate the target in mammalian models of the disease. In the case of targets associated with changes in cholesterol synthesis, a reliable, sensitive detection method is key for ensuring that the results can be interpreted with confidence. When considering cholesterol homeostasis, interpretation may be even more cumbersome given the multiplicity of cellular regulatory steps and compensatory mechanisms at play; indeed, such mechanisms can present a challenge in experimental conditions. Small variations in cell culture conditions, or other confounding factors introduced in the experimental setting, may affect the results of cholesterol level determination. One study found a link between SIRT2 and cholesterol in worm, fly and cell culture HD models 13. This link should be validated using a more sensitive method to detect cholesterol 24, 25, especially since others failed to confirm *in vivo, *in mouse models, SIRT2 inhibition as a therapeutic strategy in HD 26.

To conclude, the hydrophobicity of different lipids, their susceptibility to oxidation in different experimental conditions, the specific extraction procedures used to obtain the desired species and the technological limitations of the methods used for measuring lipid levels, all suggest that the HD scientific community should use caution in interpreting results. In addition, it may be helpful to consult with lipid experts. Our findings make it clear that the most sensitive and reliable analytical techniques, along with appropriate sample preparation, should be used for unambiguous and unbiased measurement and interpretation of cholesterol dysfunction in the context of HD.

## Methods

**Reagents.** Filipin, Nile Red, cholesterol, MβCD and simvastatin were purchased from Sigma Chemicals Co. (St. Louis, MO, USA). Filipin is unstable in solution, so a 50 mg/ml stock was prepared in DMSO and stored in aliquots at -80°C to avoid freezing/thawing. The aliquots were always protected from exposure to light. Simvastatin was prepared as an 11.94 mM stock solution in DMSO. MβCD was added to the medium to achieve the final indicated concentration.

**Cell cultures.** ST Kin^7/7Q^ and ST Kin^109/109Q^ cells, and inducible immortalized striatal cells expressing the first 548 amino acids of muHTT with 128 CAG repeats after doxycycline administration, were grown at 33°C in high glucose DMEM (Euroclone) supplemented with 10% Fetal Bovine Serum (FBS), penicillin (100 U/ml) and streptomycin (100 μg /ml) plus 2 mM L-glutamine as described elsewhere 5, 16. In the case of inducible HD cells, FBS was used that was tested to ensure that it was tetracycline-free.

**Cholesterol and MβCD treatment.** Cells were counted and seeded in 6-well multiwell plates (for cholesterol detection with the enzymatic method or by GC-MS) or in 12-well multiwell plates (for colorimetric methods) and incubated with different concentrations of cholesterol (stock solution, 10 mg/ml in ethanol) for 16 hours or with media containing 1, 5, 10 or 25 mM MβCD for 1 hour.

**Immunofluorescence staining and microscopy analysis.** For Nile Red and filipin staining, the cells were plated on coverslips and fixed in 4% paraformaldehyde for 15 min. For Nile Red staining, the cells were incubated with a solution containing 10 µg/ml Nile Red for 15 min. For filipin staining, the cells were washed with 50 mM NH_4_Cl in PBS (2 x 5 min) to quench PFA, washed once with PBS and incubated with filipin solution (100 µg/ml in PBS) for 30 min with mild agitation and protected from the light. After a wash with PBS, the cells were incubated in H_2_O (5 sec) to remove PBS salts and mounted with Mowiol. The stained cells were examined, and images were acquired with either a Leica DMI 6000B inverted microscope or a Leica 4000B upright microscope with LAS-AF imaging software. The images were processed using Adobe Photoshop.

**Measurement of total cholesterol by the enzymatic method.** The measurement of total sterol levels using the enzymatic method were performed on a lipid fraction isolated with solvent extraction as described by Valenza et al., 2005 and on protein lysate that was isolated as described previously by Del Toro et al., 2010. Briefly, for lipid extraction the cells in each well were washed twice with 1 ml cold PBS and 1 ml of hexane/isopropanol (3:2, v/v) was added to the well. After rocking at room temperature for 1 hour to extract the lipids, the liquid in each well was transferred to a glass tube, the solvents were removed using a stream of nitrogen at room temperature and the lipid pellets were solubilised. The protein lysate for sterol measurements was prepared with the same buffer as in Del Toro et al., 2010: ice-cold 10 mM Tris, pH 7.5, 150 mM NaCl, 5 mM EDTA, 1 mM PMSF, 2 mM orthovanadate, 10 ug/ml aprotinin and 1 ug/ml leupeptin. Total cholesterol was assayed in lipid extracts and protein lysates using the Amplex® Red Enzyme Assay (Invitrogen) according to the manufacturer’s instructions and normalized by protein concentration. The latter was determined using a BCA protein assay kit (Pierce, Rockford, IL, USA).

**GC-MS and isotopic-dilution mass spectrometry.** Cell homogenates were prepared in PBS, and 80 μl of homogenate was added to a screw-capped vial sealed with a Teflon-lined septum along with 5 µg of 2H6-cholesterol and 100 ng of 2H4-lathosterol (CDN Isotopes) as internal standards. To prevent auto-oxidation, 25 μl of butylated hydroxytoluene (BHT) (5 g/l) and 25 μl of EDTA (10 g/l) were added to each vial, and vials were flushed with argon to remove air. Alkaline hydrolysis was allowed to proceed at room temperature (22°C) for 1 h in the presence of 1 M ethanolic KOH solution with magnetic stirring. After hydrolysis sterols (cholesterol, lathosterol, lanosterol, desmosterol and 7-dehydrocholesterol) were extracted two times with 5 ml of cyclohexane plus 5 mL of ethyl-acetate. The organic solvents were evaporated under a gentle stream of argon and converted into trimethylsilyl ethers [BSTFA + TMCS 1%]. GC-MS was performed on a Perkin Elmer Clarus 600C gas chromatographer- mass selective detector. The GC was equipped with a DB-XLB (30 m × 0.25 mm i.d. × 0.25 μm film; J & W Scientific, Palo Alto, CA, USA), and the injection was performed in the splitless injection mode using helium (1 ml/min) as a carrier gas. The initial temperature of 180°C was maintained for 1 min, increased by 20°C/min up to 270°C, then increased by 5°C/min to the final temperature of 290°C, which was maintained for 10 min. The mass spectrometer was used in selected ion-monitoring mode, and the neutral sterols were monitored as their TMSi derivatives using the following masses: 2H6-cholesterol at m/z 464 (M+-OTMSi); cholesterol at m/z 458 (M+-OTMSi); 2H4-lathosterol at 462 m/z (M+-OTMSi), lathosterol at 458 m/z (M+-OTMSi), desmosterol at m/z 343 (M+-OTMSi); 7-dehydrocholesterol at m/z 325 (M+-OTMSi) and lanosterol at 393 and 498 m/z (M+-OTMSi). Peak integration was performed manually, and the sterols were quantified from the selected-ion monitoring analyses by comparison with internal standards using standard curves. The identity of all of the sterols was verified by comparison with the full-scan mass spectra of authentic compounds. Additional qualifier ions (characteristic fragment ions) were used for structural identification.

**Ca2+ measurements with recombinant aequorin.** Before transfection, cells were seeded onto 13-mm cover glass slips for the aequorin (AEQ) measurements and allowed to grow to 70–80% confluence. The cells were transformed using the Ca2+-phosphate technique in the presence of 1.5 μg AEQ cDNA. The Ca2+ measurements were carried out 48 hours after transfection. To reconstitute AEQ, the coverslips with the transfected ST Kin^7/7Q^ cells were incubated for 65 minutes with 5 mM coelenterazine in Krebs-Ringer buffer (KRB) medium with 1 mM CaCl2 and then transferred to the perfusion chamber of a low-noise photomultiplier with a built-in amplifier-discriminator (Thorn-EMI photon counting board). All the AEQ measurements were carried out at 37°C in modified KRB (135 mM NaCl, 5 mM KCl, 0.4 mM KH_2_PO_4_, 20 mM HEPES, 0.1% glucose; pH 7.4). Each experiment began when the cells were perfused with 1 mM CaCl2. After about 30 s, cells were perfused with Mg^2+^-free KRB containing CaCl_2_ (1 mM), glutamate (100 μM) and glycine (100 μM) or NMDA 100 μM (Invitrogen). After 120 s, ATP (100 μM) was added to elicit transient Ca^2+^ efflux. At the end of each experiment, cells were lysed by adding 100 μM digitonin in a hypotonic Ca2+-rich solution (10 mM CaCl2) to discharge the remaining unused AEQ pool. The light signal was collected by the photomultiplier and stored on an IBM-compatible computer and off-line calibrated into Ca2+ concentration values. Luminescence was calibrated off-line into Ca2+ concentration values using a computer algorithm based on the Ca2+ response curve of wild-type aequorin.

**RNA isolation and PCR measurements.** Twenty-four hours after plating, AEQ-transfected and untransfected cells were harvested for RNA isolation using Trizol (Invitrogen). RNA was isolated, purified and reverse transcribed using a commercially available kit (Invitrogen). The cDNA was subjected to real time PCR amplification (CFX96, Biorad) as described in Valenza et al., 2010. PCR was carried out with the same quantity of cDNA using the following primers: NMDA subunit NR1 for: GCCTCCAGCTTCAAGAGACGT; rev: TGTGTTCCCGTCATAGGGAGAG. mGLUR1 for: GGTCCCTTCTGACACTTTGC; rev: CATTCCACTCTCGCCGTAAT. mGlUR5 for: GCCATGGTAGACATAGTGAAG; rev: TAAGAGTGGGCGATGCAAAT. NMDA 2B for: ACGGCAGCAAATCCTACTTCT; rev: ACCACTGGCTTATTGGTGACA. GAPDH for: CAAGGTCATCCATGACAACTT; rev: GGGCCATCCACAGTCTTCTG.

**Statistical analysis.** Graphpad Prism v.4.0 (Graphpad software, San Diego, CA, USA) software was used to perform one-way ANOVA and Newman-Keuls Multiple Comparison post-hoc testing.

## Competing Interests

The authors have declared that no competing interests exist.

## Authors' contributions

MM and MV performed most of the experiments and contributed to the conception and design of the experiments and the acquisition, analysis and interpretation of data; VL and CC performed all the experiments involved mass spectrometry, and VL and SDD contributed to analysis and interpretation of mass spectrometry data; CS, ADM, ECar performed all the experiments related to NMDA and contributed to analysis and interpretation of data; ECatt coordinated the study, made contribution to conception and design, to analysis and interpretation of data. MV, MM and ECatt wrote the paper.

*MM and MV contributed equally to this work.

## Figures and Tables

**(Fig. S1) Quantification of images of filipin staining in ST Kin cells.**
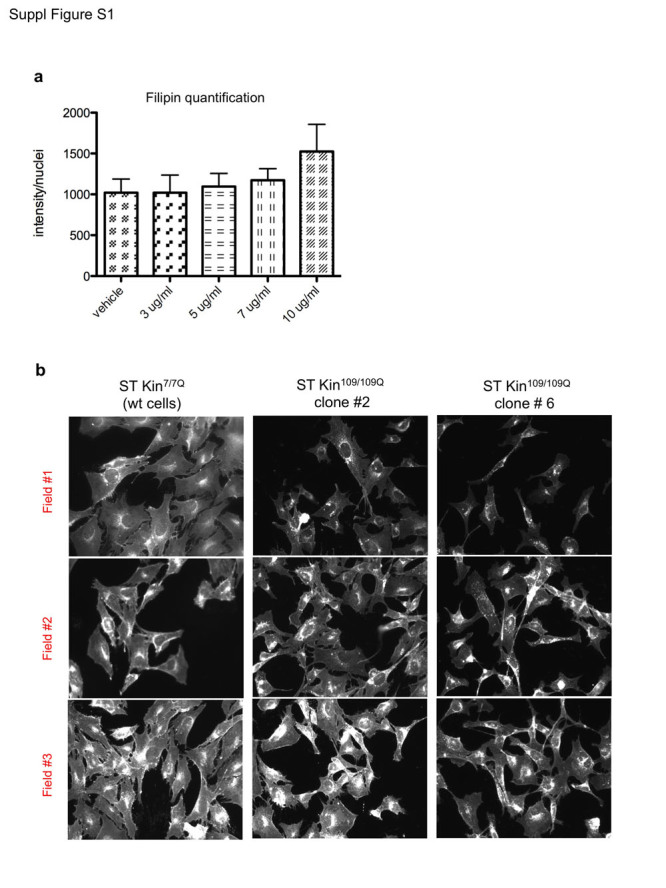
Quantification of filipin staining was performed using imageJ software. a) Ten fields were analysed for each condition. The graph represent the average of the quantifications ± SEM. b) Representative fluorescent images of filipin-stained cells showing the high variability in the fluorescent intensity in different fields of the same well for each cell line. The images shown were cropped from 20X magnification images.

**(Fig. S2) NMDA activity in ST Kin cells.**
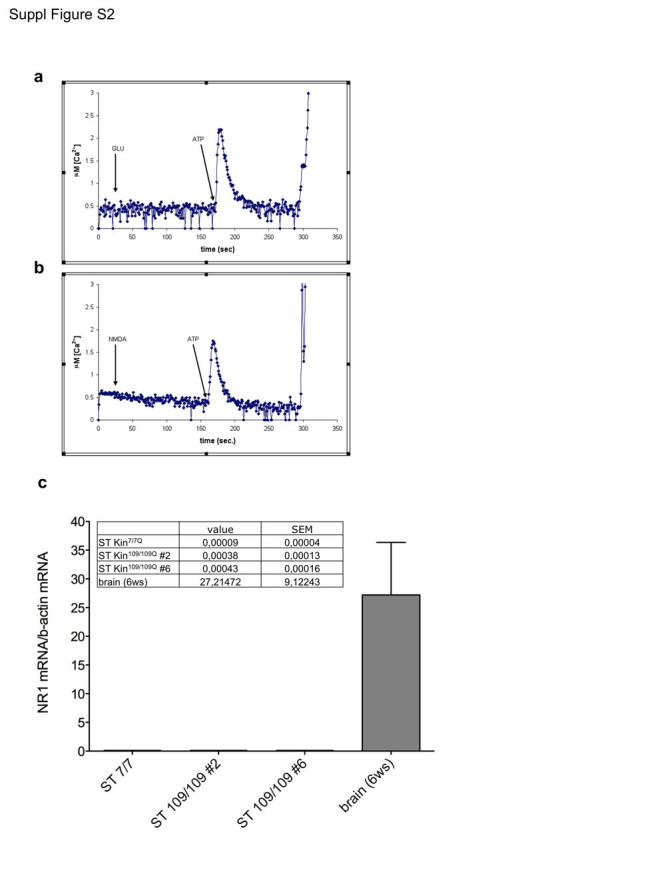
Ca2+ transients in the cytosol of ST Kin^7/7Q^ cells were induced by stimulation with the purinergic receptor agonist ATP. Cell culture and transfection protocols and methods for monitoring cytosolic Ca2+ using AEQ are described in the Materials and Methods. a) Effects of adding glutamate (GLU) and ATP. b) Effects of adding NMDA and ATP. The opening of the InsP3 receptor, which is induced by stimulation of the purinergic receptors, elicits the expected Ca2+ transient, whereas no transient is elicited by the addition of glutamate or NMDA. c) Quantification of NR1 mRNA levels in striatal knock-in cells. The NMDA subunit NR1 is not expressed in ST Kin^7/7Q^ cells (0.00009 ± 0.00004) or ST K-in^109/109Q^ cells (clone #2, 0.00038 ± 0.00013; clone #6, 0.00043 ± 0.00016). The grey column represents the expression of NR1 mRNA as a positive control (levels in brains from 6-week-old mice: 27.21472 ± 9.12243).
